# Is suturing of the bladder defect in benign Enterovesical fistula necessary?

**DOI:** 10.1186/s12893-019-0542-4

**Published:** 2019-07-08

**Authors:** Ł. Dziki, M. Włodarczyk, A. Sobolewska-Włodarczyk, M. Mik, R. Trzciński, A. G. Hill, A. Dziki

**Affiliations:** 1 Department of General and Colorectal Surgery, Medical University of Łódź, Łódź, Poland; 2 Department of Biochemistry, Medical University of Łódź, Łódź, Poland; 3 Department of Surgery, Middlemore Hospital, University of Auckland, Private Bag 93311 Otahuhu, Auckland, New Zealand

**Keywords:** Enterovesical fistula, Urinary bladder, Fistula, Colectomy, Colovesical fistula

## Abstract

**Background:**

Enterovesical fistula (EVF) is a abnormal connection between the intestine and the bladder. The aim of the study was to analyze whether closure of the defect in the bladder wall during surgery is always necessary.

**Methods:**

Fifty-nine patients with benign EVF undergoing surgical treatment were enrolled. A one-stage surgical procedure was performed in all patients. After the separation of the diseased bowel segment, methylene blue was introduced. Through a catheter into the bladder. Only patients with urinary bladder leakage were sutured.

**Results:**

The most common intestinal fistula involving the urinary bladder was colovesical fistula, observed in 53% of cases. Two-thirds of patients had diverticular disease as the underlying pathology. There was no relationship between suturing of the bladder and perioperative complications. Recurrent EVF was observed in one patient with bladder suturing and in two patients without suture.

**Conclusions:**

These findings suggest that closure of the bladder defect is not necessary in cases where a leak is not demonstrated from the bladder intraoperatively. This study is limited by its retrospective design and small numbers and a randomized controlled trial is recommended to answer this question definitively.

## Background

Enterovesical fistula (EVF) is characterized by an abnormal communication between the bladder and the intestine. EVF most commonly occurs due to malignancy or inflammatory diseases [1, 2]. Although EVF is relatively rare it causes significant morbidity and may noticeably affect the quality of life of the patient. A diagnosis of EVF can be challenging and is often delayed for several months after symptoms begin. Radiological imaging plays a crucial role in establishing the site, course, and complexity of fistulae, and in identifying the etiological factor [3, 4].

EVF is classified based on the bowel segment that is involved in the abnormal communication with the bladder. Colovesical fistula is the most common form, and is most frequently located between the sigmoid colon and the dome of the bladder [5, 6].

Management of EVF depends mainly on the underlying pathology, the site of the bowel lesion, and the preoperative clinical status of the patient. The most frequently used operative method is to resect the offending bowel segment and to close the bladder. However, it is not clear whether closure of the defect in the urinary bladder wall is always required [7, 8]. It has been reported that simple resection of the affected intestine is sufficient to heal the defect and repair of the bladder may not be required [9–12].

This study investigates the most appropriate surgical treatment approach to management of the defect in the urinary bladder.

## Methods

### Patients

This paper is reported according to the Strengthening the Reporting of Observational Studies in Epidemiology (STROBE) guideline. This study was conducted with the ethical principles of the 1975 Declaration of Helsinki and approved by the Committee of Bioethics of Medical University of Lodz (RNN/578/12/KB). Written informed consent was obtained from all patients. This retrospective study was conducted in adult patients who underwent a surgical procedure for the treatment of EVF between January 2008 and December 2015 at the Department of General and Colorectal Surgery of the Medical University of Lodz, Poland. The existence of EVF was diagnosed and confirmed by at least one of the following diagnostic modalities: cystoscopy, colonoscopy, cystography, computed tomography (CT) and magnetic resonance imaging. Patients with hepatic disorders, neoplastic diseases and those who underwent radio- or chemotherapy were excluded from the study. Fifty-nine EVF patients with benign disease (32 men and 27 women; mean age ± standard deviation 58.7 ± 17.1 years), who underwent surgical treatment of EVF were included in the final analysis.

### Surgical treatment of benign EVFs

A standardized surgical approach was applied for all patients; open surgery with a one-stage procedure was performed with resection and anastomosis. After the offending bowel segment was separated, the urinary bladder, was filled with 350 ml of methylene blue and the bladder wall defect was closed with single sutures in patients with leakage, while no suturing was performed in patients without leakage. In all patients, drainage of the bladder by a Foley catheter was maintained for seven days after the operation, along with antibiotic treatment (metronidazole and ciprofloxacin).

### Data collection

The records were reviewed retrospectively and were recorded onto a standardized case report form that included demographic and anthropometric data, the type of fistula, existing symptoms, the primary disease process, laboratory data, surgical management and treatment outcome. All data were transferred to a database for further analysis. Follow-up information was obtained by out-patient-clinic records.

### Statistical analysis

The data were analyzed using Statistica 12.5 software (StatSoft, Inc., United States). Results were expressed as mean ± standard deviation. The Shapiro-Wilk test was used to test the distribution of variables. Comparisons between groups were performed using the Student’s t-test and non-parametric data were compared using the Mann-Whitney U-test and the χ2 test. A result of *p* < 0.05 was considered significant.

## Results

Fifty-nine patients were confirmed with benign EVF, including thirty-two males. The mean age of the patients was 58.7 ± 17.1 years. The most common EVF was colovesical fistula, observed in 31/59 cases (53%). The baseline characteristics are presented in Table 1.

**Table 1:** Baseline demographic characteristics of patients with enterovesical fistula (EVF) enrolled in the final analysis

		Patients with EVF	
		group with the bladder suturing	group without sutures
Gender	women, *n*	14	13
	men, *n*	17	15
Age, years		57.7 ± 19.2	59.1 ± 20.1
BMI, kg/m^2^		24.1 ± 6.1	27.4 ± 4.8
Type of fistula	Colovesical, *n* (%)	19 (32.3%)	12 (20.3%)
	Ileovesical, *n* (%)	8 (13.5%)	7 (11.9%)
	Rectovesical, *n* (%)	6 (10.2%)	5 (8.5%)
	Appendicovesical/Caecovesical, *n* (%)	1 (1.7%)	1 (1.7%)
Primary disease	Diverticulitis, *n* (%)	11 (18.6%)	10 (16.9%)
	Crohn’s disease, *n* (%)	16 (27.1%)	16 (27.1%)
	Other, *n* (%)	3 (5.1%)	3 (5.1%)

Data are presented as mean ± standard deviation or number (percentage)

*BMI*-body mass index

The most common primary disease was diverticular disease, recorded in 21 patients (68%). In 29% cases, (9/31) the colovesical fistula was related to Crohn’s disease and in one case, to a previous operation (1/31; 3.2%). One case of appendicovesical fistula was observed in the study as complication of appendicitis. The caecovesical fistula was in a patient with ileocecal Crohn’s disease. In all patients with ileovesical fistula, the primary disease related to the development of EVF was Crohn’s disease. In all patients with Crohn’s disease the past administration of corticosteroid was comparable in both groups with and without urinary bladder suturing (2/7 vs 3/8).

Dysuria and recurrent urinary tract infections were observed in all patients. The other common symptoms of EVF were pneumaturia and faecaluria, noted in 66% (39/59) and 46% (27/59) of patients respectively. Abdominal pain as the leading symptom of EVF was observed in 10 patients (17%). Two patients with rectovesical fistula (18%) presented with urinary leakage from the rectum.

In the present study, all patients underwent CT and colonoscopy to confirm the diagnosis of EVF and to assess fistula involvement. Other diagnostic tests used for evaluation of the presence of EVF are listed in Table 2. In this study CT scan confirmed the presence of the fistula tract in all cases.

**Table 2:** Diagnostic studies used for the evaluation of the presence of enterovesical fistula

Type of study	Subjects, *n* (%)
CT Scan	59 (100%)
Colonoscopy	59 (100%)
Cystoscopy	42 (71.2%)
Cystography	25 (42.4%)
Lower GI Series	5 (8.5%)
Fistulography	2 (3.4%)

The median length of bowel resection was 12 cm (7-31 cm). A primary open surgical single stage procedure with bowel anastomosis, without protective stoma, was performed in all patients.

Urinary bladder leakage of Methylene blue was observed in 31% of EVF patients (18/59). In all patients with leakage, the urinary bladder wall defect was closed with a single No. 0 absorbable polyglactin 910 (Vicryl) suture. Postoperative pathological evaluation of surgical specimens confirmed benign disease in all patients as the underlying cause of the EVF.

Major post-operative complications requiring surgical intervention were observed in only one patient (5.6%; 1/18) with bladder suturing and one patient (2.4%; 1/41) in the group without sutures (p = ns); both related to local sepsis in the pelvis related to anastomotic leakage. Anastomotic leakage in both cases was confirmed by CT scanning with rectal contrast and successfully managed using a conservative approach including bowel rest, nutritional therapy and antibiotics. No other major post-operative complications or perioperative mortality occurred in either group. No significant differences were observed between the groups with regard to the occurrence of wound infection (5.6% vs. 4.9%; *p* = 0.896).

No bladder leakage was observed postoperatively in either group. During a median follow-up of 48 months (range 3 to 103), recurrent EVF was observed in one patient with urinary bladder suturing and in two patients without suture (*p* = 0.913). All cases of recurrent EVF were observed in patients with Crohn’s disease. The recurrent EVF was repaired successfully by a second operation in two of these cases, and a third surgical operation was required in one subject. All cases of iatrogenic EVF caused by previous bowel surgery were successfully managed using a one-stage repair without recurrence. No difference in post-operative outcome was found with regard to the management of iatrogenic EVF in patients with or without bladder suturing.

## Discussion

EVF is an uncommon complication of both malignant and benign disorders and surgery remains the treatment of choice. The decision to close the bladder defect at the time of surgery in benign EVF is still controversial, and our findings indicate that bladder suture is probably not necessary in the situation where no leakage of methylene blue occurs from the bladder on testing.

Most patients with EVF will need surgical intervention [2]. The surgical technique involves blunt dissection of the bowel from the bladder, resection of the intestine and primary anastomosis. As the fistulous tract opening in the bladder may not be directly visible, distention of the bladder with methylene blue solution instilled through a catheter may be a helpful indicator of a leak [4].

Intraoperative closure of the bladder defect remains controversial. The literature is replete with case reports that have described different surgical approaches to treatment of EVF. Such reported methods include the blunt ‘pinch off’ technique of separating the colon and the bladder, followed by simple closure of the bladder defect. Others describe an omental patch to close the bladder defect or include resection of the involved defect, with closure of the vesical bladder using absorbable sutures and a protective omental flap [13–15]. However, no clinical trials have been performed which identify the most appropriate method.

Recent studies suggest that the laparoscopic approach is a safe option for the treatment of EVF of benign inflammatory origin. In laparoscopy, operative treatment involves separation of the inflammatory mass and resection of the affected colorectal segment. However a high conversion rate (up to 30%) has plagued the laparoscopic approach due to difficulties with the resection and bladder closure [16–18]. Our series was entirely open but as laparoscopy matures in colorectal surgery in Poland this is likely to become our preferred approach.

Although the symptoms associated with fistulas involving the urinary bladder are distinctive, confirmation of the diagnosis and identification of the anatomy of the fistulous track are difficult. CT, cystoscopy, endoscopy, barium enema, and cystography are currently used for EVF diagnosis [10, 19–21]; however, in some cases, these imaging techniques cannot precisely demonstrate the fistula. In our study CT scans demonstrated the presence of the fistula tract in all cases.

This study is limited by its small size, its retrospective and non-randomised nature and the inclusion of only patients with benign disease. Further, the catheter was left in for a week following surgery which is standard practice. Finally, the question of whether suturing is required at all even when there a bladder leak is demonstrated is not answered by this study and further studies may wish to consider this issue.

The findings of this study have been presented as an abstract at the 1st Ambroise Paré International Military Surgery Forum (APIMSF) Congress [22] and as a poster at the United European Gastroenterology Week 2016 [23].

## Conclusions

EVF is an uncommon complication of both malignant and benign disorders and surgery remains the treatment of choice. The decision to close the bladder defect at the time of surgery in benign EVF is still controversial, and our findings indicate that bladder suture is probably not necessary in the situation where no leakage of methylene blue occurs from the bladder on testing. An appropriately powered randomized clinical trial would be useful to answer this question definitively.

## Data Availability

The data that support the findings of this study are available from the corresponding author upon reasonable request.

## Abbreviations

BMI: Body Mass Index
CT: Computed Tomography
EVF: Enterovesical Fistula
